# Sewage Systems Surveillance for SARS-CoV-2: Identification of Knowledge Gaps, Emerging Threats, and Future Research Needs

**DOI:** 10.3390/pathogens10080946

**Published:** 2021-07-28

**Authors:** Fatemeh Amereh, Masoud Negahban-Azar, Siavash Isazadeh, Hossein Dabiri, Najmeh Masihi, Mahsa Jahangiri-rad, Mohammad Rafiee

**Affiliations:** 1 Environmental and Occupational Hazards Control Research Center, Shahid Beheshti University of Medical Sciences, Tehran 35511, Iran; amereh@sbmu.ac.ir (F.A.); najmehmasihi72@gmail.com (N.M.); 2 Department of Environmental Health Engineering, School of Public Health and Safety, Shahid Beheshti University of Medical Sciences, Tehran 35511, Iran; 3 Department of Environmental Science and Technology, University of Maryland, College Park, MD 20740, USA; 4 Environmental Service, Suez Water North America, Paramus, NJ 07652, USA; siavash.eisazadeh@gmail.com; 5 Department of Medical Microbiology, School of Medicine, Shahid Beheshti University of Medical Sciences, Tehran 35511, Iran; h.dabiri@hotmail.com; 6 Water Purification Research Center, Tehran Medical Sciences, Islamic Azad University, Tehran 19168, Iran; m.jahangiri@iautmu.ac.ir

**Keywords:** COVID-19, SARS-CoV-2, virus concentration/enrichment methods, virus detection/quantification, wastewater-based epidemiology

## Abstract

The etiological agent for novel coronavirus (COVID-19, Severe Acute Respiratory Syndrome Coronavirus 2 (SARS-CoV-2), not only affects the human respiratory system, but also the gastrointestinal tract resulting in gastrointestinal manifestations. The high rate of asymptomatic infected individuals has challenged the estimation of infection spread based on patients’ surveillance, and thus alternative approaches such as wastewater-based epidemiology (WBE) have been proposed. Accordingly, the number of publications on this topic has increased substantially. The present systematic review thus aimed at providing state-of-the-knowledge on the occurrence and existing methods for sampling procedures, detection/quantification of SARS-CoV-2 in sewage samples, as well as anticipating challenges and providing future research direction to improve the current scientific knowledge. Articles were collected from three scientific databases. Only studies reporting measurements of virus in stool, urine, and wastewater samples were included. Results showed that improving the scientific community’s understanding in these avenues is essential if we are to develop appropriate policy and management tools to address this pandemic pointing particularly towards WBE as a new paradigm in public health. It was also evident that standardized protocols are needed to ensure reproducibility and comparability of outcomes. Areas that require the most improvements are sampling procedures, concentration/enrichment, detection, and quantification of virus in wastewater, as well as positive controls. Results also showed that selecting the most accurate population estimation method for WBE studies is still a challenge. While the number of people infected in an area could be approximately estimated based on quantities of virus found in wastewater, these estimates should be cross-checked by other sources of information to draw a more comprehensive conclusion. Finally, wastewater surveillance can be useful as an early warning tool, a management tool, and/or a way for investigating vaccination efficacy and spread of new variants.

## 1. Introduction

During the years 2003–2004, the outbreak of severe acute respiratory syndrome coronavirus (SARS-CoV) afflicted people throughout the world and was poised to be the next pandemic. The virus, however, was responsible for just over 8400 cases with fewer than 1000 deaths in 29 countries and studies concerning infection-control measures were instrumental in severely curtailing the potential for a pandemic [1,2]. However, an outbreak of pneumonia of unknown etiology first reported in Wuhan (Hubei province, China) in the late 2019, has startled the world and caused the globe to undergo unprecedented change in a short space of time. Metagenomics sequencing of broncho alveolar lavage samples shed light on the association of this outbreak with a novel coronavirus (nCoV) [2]. The “novel coronavirus-infected pneumonia” was officially designated as SARS-CoV-2 after being provisionally named as 2019-nCoV [3,4]. On 31 January 2020, the World Health Organization (WHO) declared the outbreak to constitute a public-health emergency of international concern when the disease was reported in 114 countries and thence named this disease as Corona Virus Disease 2019 (COVID-19) on 11 February 2020 [5,6]. The status of the outbreak was upgraded from epidemic to pandemic on 11 March. By 2 July 2021, 182,653,642 confirmed cases, including 3,955,835 deaths, was officially announced all over the world, with distressing consequences on human health and economy, particularly in the United States, India, Brazil, and Russia, among others [7].

Current evidence for COVID-19 is primarily confined to flu-like symptoms with most affected patients suffering from fever, dry cough, and difficulty in breathing but some become seriously ill and even die [8,9]. The available epidemiological evidence strongly suggests that COVID-19 is primarily transmitted through respiratory droplets and contact routes [10]. Recent tracing of SARS-CoV-2 genetic material—viral RNA—in stool and urine of hospitalized COVID-19 patients [11,12,13], however, shed light on the rapidly evolving picture of this new disease. This pattern of spread is highly suggestive of virus dissemination by aqueous matrices, and the circulation of virus was speculated to be occurred from malfunctioning sewage works (sewer networks and wastewater treatment plants (WWTP)) in the community [14,15]. Sanitary facilities have been hypothesized as possible amplifiers and disseminators of the virus [16]; the pathogenic agent, most likely acquired from infected patients, may have released in their excreta (i.e., saliva, sputum, and feces) and then possibly becoming waterborne. Nonetheless, neither WHO nor the US Centers for Disease Control and Prevention (CDC) still don’t consider COVID-19 as waterborne and finding clues to support this claim throughout literature has not reached a clear conclusion.

The scientific community has recently witnessed an interest in shedding of virus into feces as well as the presence and persistence of SARS-CoV-2 in health care and municipal effluents and thus the potential of sewage to spread COVID-19 [15,17]. Outbreak investigations strongly suggest that infections in the hospital and the community may occur through this route. The presence of SARS-CoV-2 (either viable virus or associated viral debris) in feces indeed raises the potential for sewage analysis in order to inform epidemiological monitoring of COVID-19 as a complementary approach for current clinical surveillance techniques by providing information on the prevalence and spread of disease in a population [18]. The method as a relatively new approach in public health, based on raw wastewater fingerprinting to obtain qualitative and quantitative data within a given wastewater catchment, not only provides an early warning sign for disease outbreaks but also acts as a smart way of imposing preemptive quarantine [19].

There is wide-ranging research into sewage monitoring of SARS-CoV-2 often with two overarching objectives: (1) to detect the presence/prevalence of virus in a population; and (2) to assess infection risk to the public and sewage workers/operators from untreated/partially treated contaminated sewage and effluent as well as air surrounding sewage work facilities. As an affordable, convenient, and practical program, monitoring viral RNA in sewage can also be used to complement the current clinical surveillance by providing information on the prevalence and spread of disease in a population. The present review sets out to outline an overview of the current knowledge addressing the occurrence of SARS-CoV-2 in the feces of affected individuals and its dissemination in the public sewage system. The goal is to recognize key concepts, research gaps, and types of evidence within the following topics: (i) wastewater/effluent sampling strategy [20] methods for the detection and quantification of SARS-CoV-2 RNA in wastewater, particularly concerning an evolving field of wastewater-based epidemiology (WBE); and (ii) the potential ecological risks (secondary transmission risk) to the wider environment. We tried to highlight the areas where further research is needed and made further recommendations by interpreting current state of knowledge dealing with these topics.

## 2. Methods

A systematic review was carried out to synthesize our current knowledge of the presence of SARS-CoV-2 both in stool of confirmed patients and wastewater. The Preferred Reporting Items for Systematic Reviews and Meta-Analyses (PRISMA) statement was used to report the results of our review.

Our search was conducted across the Scopus, PubMed, and Web of Science databases for relevant literature with no restriction for publication date or language. The search included the keywords: wastewater OR sewage OR effluent OR fec* OR faec* OR diarrh* OR meconium* OR melena OR stool OR urine AND SARS-CoV-2 OR 2019 nCoV OR COVID-19. To give a comprehensive view of the topic, the literature search was extended to preprints using the medRxiv server (https://www.medrxiv.org/ (accessed on 28 December 2020)), with the same criteria. Searches were conducted on 28 December 2020. MeSH terms were applied when using the PubMed database in order to employ the thesaurus of medical vocabulary. Article inclusion required confirmation of infection via stool sampling, and/or confirmation of viral wastewater contamination.

**Figure 1:**
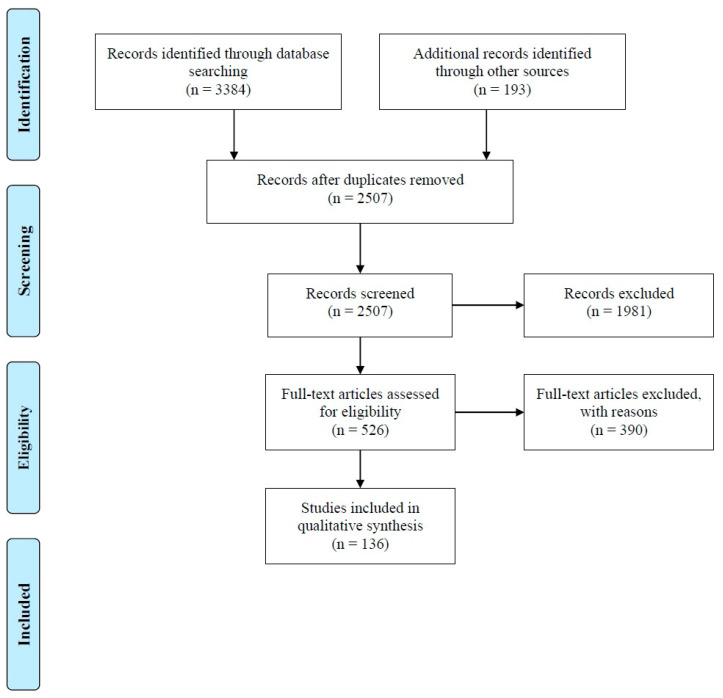
Flow chart for the systematic literature search.

A total of 3384 potentially appropriate articles was identified via the first review phase, decreasing to 2507 upon deduplication using the Mendeley Reference Manager Software (Figure 1). The screening phase was undertaken through an assessment of article title and abstract, resulting in 526 articles going forward for eligibility assessment. Full texts were independently and concomitantly analyzed by two researchers (FA and NM), again using developed inclusion/exclusion eligibility criteria. Discrepancies between pairs of reviewers were resolved through consensus or a third adjudicator. The primary inclusion criterion was the presence of RNA or infectious (viable) SARS-CoV-2 in feces or urine of a confirmed patient and/or wastewater. Overall, upon completion of the review process, 138 articles were included for data extraction and analysis (Table 1 and Table 2). Altogether, this systematic review aims at outlining the used protocols for virus sampling, sample preparation (i.e., concentration/enrichment procedures), and analysis (i.e., target gene regions) in feces, untreated wastewater, and effluents tested in the peer-reviewed literature in order to identify actual challenges, allowing us to suggest potentially appropriate procedures and solutions. In addition, the potential risks for wastewater operators, population size normalization, and practical use of wastewater surveillance are also presented with elaborated conclusions.

## 3. Results and Discussion

**Figure 2:**
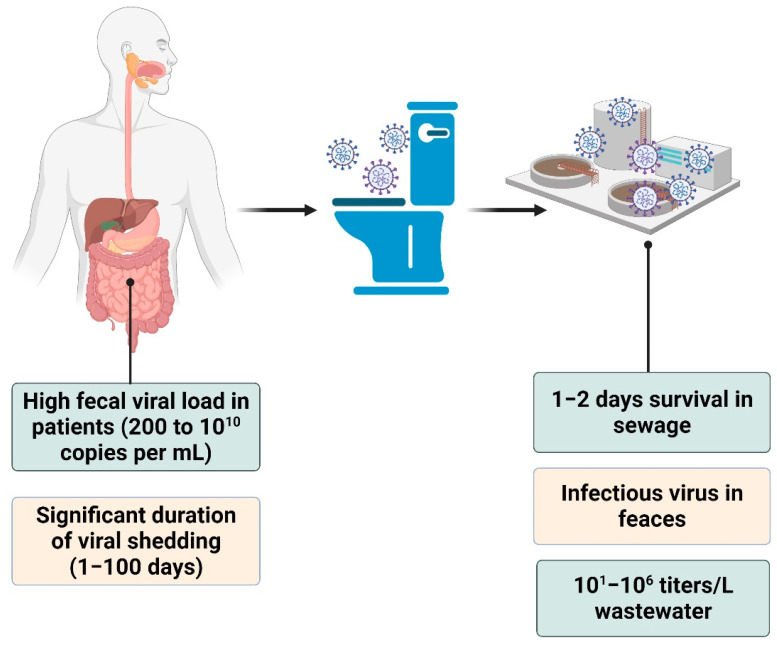
Fecal shedding of SARS-CoV-2.

From the identified studies, 88 reported on the presence of SARS-CoV-2 in feces and urine, and the remaining 48 were concerned with presence of the virus in wastewater. The PRISMA flow diagram, presented in Figure 1, demonstrates the results of our review. Figure 2 summarizes the findings of our study. The obtained results are presented and critically discussed based on the collected information in five subsections. The first two subsections contain information regarding the presence of SARS-CoV-2 in feces and wastewater (Section 3.1 and Section 3.2). Detection and quantification of SARS-CoV-2 in wastewater coupled with their accompanied challenges, potential risks, and application of results have been outlined in the third, Section 3.3. The last two, Section 3.4 and Section 3.5, present knowledge gaps and concluding remarks derived from analyzing the outcomes.

### 3.1. Gastrointestinal Manifestations and Presence of SARS-CoV-2 in Feces

The fecal shedding of SARS-CoV-2 from not only symptomatic but also asymptomatic, pre-symptomatic, and post-symptomatic COVID-19 patients has been reported [11]. Nevertheless, few studies have specifically dealt with gastrointestinal malfunctions in infected individuals and the results are divergent. To go further in our understanding, it was previously reported that respiratory manifestations precede/tail gastrointestinal symptoms in human coronavirus strains, including SARS-CoV and MERS-CoV [151]. The most frequently addressed clinical manifestations, as can be seen in Table 1, were reported in 21 studies and are categorized as abdominal pain, diarrhea, and vomiting, with diarrhea as the most frequently reported gastrointestinal malfunction. A number of studies in patients with COVID-19 underplayed the relative importance of gastrointestinal symptoms and, notably, viral shedding in stool [46,152].

In an attempt to provide an explanation, Zang et al. [153] pointed to the inactivation of SARS-CoV-2 by simulated human colonic fluid. Inactivation of infectious viral particles by digestive enzymes has also been reported previously [154]. It has been universally demonstrated that the lysis of the viral envelope in enveloped viruses such as coronaviruses results in the loss of functional receptors required for infection of susceptible cells. Nonetheless, evidence regarding the effects of digestive enzymes on the survivability of enveloped viruses in gastrointestinal tract is not conclusive. Migration to throat tissues and passage through the stomach is conceivable given that SARS-CoV-2 can survive the extreme pH of the gastric tissues and infection could then expand into the intestines where Angiotensin-converting enzyme 2 (ACE2) levels are high [155]. ACE2 is a cellular receptor that is well expressed in the respiratory tract and interacts with the spike protein to facilitate entry of virus into the host cell [5,156]. The upper oesophagus and stratified epithelial cells as well as absorptive enterocytes of the ileum and the colon have also showed highly positive for ACE2 receptor, enumerating the gastrointestinal system as a potential transmission route for SARS-CoV-2 infection [5,157]. Some studies have also reported that human coronaviruses replicate in the gastrointestinal tract [157,158]. Wang et al. reported the occurrence of an increasing rate of diarrhea in confirmed COVID-19 patients [159]. A recent review on the pathogenesis, epidemiology, prevention, and management of diarrhea during COVID-19 infection also reported an incidence rate of diarrhea ranging from 2 to 50% of cases, with an overall percentage of diarrhea onset of 10.4% which may precede or tail respiratory symptoms [160]. In support of these findings, about 2 to 10% of hospitalized COVID-19 patients have been reported to have diarrhea [34,159,161,162,163,164]. Meanwhile, in an analysis of data from the Hong Kong cohort of patients with COVID-19, Cheung et al. [152] reported that 25.4% of affected individuals had diarrhea among other gastrointestinal symptoms. The same research in a meta-analysis of 60 studies, comprising 4243 patients, demonstrated that the prevalence of all gastrointestinal manifestations was 17.6%. These observations are in line with reported SARS-CoV pattern in 2003, where 16–73% of patients had chronic diarrhea during the first week of the illness while presenting pulmonary symptoms [165,166]. Thus, although some discrepancies between studies exist, they are consistent with those of official data from WHO pointing to that between 2 and 27% of COVID-19 infected individuals have diarrhea [167]. More studies of comparable quality are needed to conclude the rate of diarrhea in COVID-19 cases with severe, mild, or no symptoms.

In total, 88 studies dealing with the shedding of SARS-CoV-2 in stool were retrieved (Table 1). Although data on the subject is still in its infancy, there is a growing evidence for the presence of SARS-CoV-2 genetic material in human feces [168], detected mainly through reverse transcription polymerase chain reaction (RT-PCR) or reverse transcription quantitative real-time PCR (RT-qPCR) following RNA isolation [169]. Nevertheless, it is worth mentioning that there are small differences in protocols for virus isolation from feces between laboratories. Positive isolation of SARS-CoV-2 RNA from hospitalized patient’s stool samples or their rectal swabs was reported in 48% (31/65) [46], 53% (39/73) [80], and 67% (6/9) [64] of cases, while the virus RNA has also been detected in human feces of at least 82% (55/66) [79] of tested patients in a recent study in China. Nonetheless, few studies have investigated the fecal shedding in cases with mild symptoms or even asymptomatic individuals [170]. Preliminary estimates of asymptomatic infected individuals range from 17.9 to 30.8% [156]. Also, the persistence of fecal shedding in affected patients and its association with respiratory shedding largely remains elusive. It is somewhat surprising that virus RNA has been detected in stool samples from patients with COVID-19 even in samples collected after respiratory samples tested negative [51,80], demonstrating that shedding of virus RNA into feces lasts long after its clearance from pulmonary tract. Although it is unclear how long the fecal shedding continues, Jiang et al. have reported that SARS-CoV-2 was detectable in stool samples of an asymptomatic infected case for as many as 42 days [171]. Surprisingly, viral detection of SARS-CoV-2 in nasopharyngeal samples of infected case in this study was negative. Several studies on stool samples, in agreement with this finding, reported detection of SARS-CoV RNA from the fifth day, demonstrating a peak in viral titer at the 11th day which lasted till the 30th day [172]. It is encouraging to compare these records with that found by Leung [151] who reported that gastrointestinal symptoms were followed by fecal shedding of SARS-CoV in patients with SARS up to 73 days. Even though gastrointestinal manifestations in affected cases and particularly fecal shedding of virus may not be clinically significant, there is an underlying assumption that such an information may reflect the virus circulation among human populations at this stage. Further, the dynamics and duration of fecal shedding have important implications for normalization of data, which is discussed in Section 3.5. The main application of wastewater monitoring for SARS-CoV-2 is to infer infection rates in the population in a specific sewage catchment area. The measured viral load at the influent of each WWTP reflects a cumulative amount shed by affected individuals during the relevant window—approximately 22 days with an interquartile range between 17 and 31 days, as pointed out by Zheng et al. [62]. Thus, it can be deduced that the measured concentration accounts for ill individuals—those that have and have not been clinically identified—infected several days or weeks prior to the sampling event.

**Figure 3:**
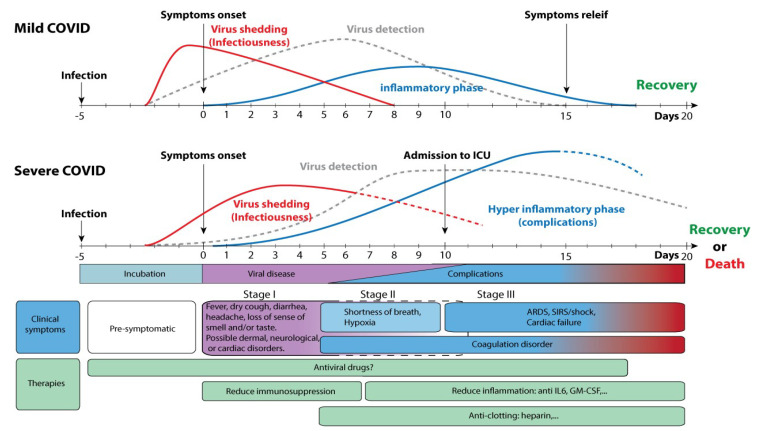
Timeline for SARS-CoV-2 shedding and detection (adapted from [173]).

The discrepancy between studies on the presence of SARS-CoV-2 viral RNA may however be assigned to the limited resources in early reports for detection which were only provided to those patients with severe respiratory symptoms. Temporal dynamics and the persistence of viral shedding in stool as provided by ViralZone [173] has been summarized in Figure 3. This figure depicts data on the onset and duration that affected individuals can discharge the virus in their stool. It is evident that gastrointestinal infections precede the development of fever and respiratory symptoms in some infected individuals, particularly in patients admitted into intensive care.

Indeed, there is only limited and, of course, heterogeneous evidence for the amount and dynamics of SARS-CoV-2 shedding in human feces. It was reported that the concentration of enteric viral particles per gram of stool during diarrhea falls in the range of 10^10^-10^12^ [174]. Also, the literature already includes several reports of SARS viral loads, pointing towards 10^6.1^ gene copies per gram (gc/g) of feces and 10^1.3^ gc/mL of urine [175]. For instance, Woelfel and coworkers [176] in their study on a cluster of nine cases highlighted that SARS-CoV-2 RNA loads could be as high as 10^7^ gc/g of stool following the onset of manifestations, which decreased to 10^3^ copies/g three weeks after the emergence of symptoms. Likewise, 1.7 × 10^6^–4.1 × 10^7^ gc/mL appears in the Han et al. [57] report, while another recent study conducted in Europe reported 6.3 × 10^6^–1.26 × 10^8^ gc/g of stool [27]. In the context of these studies, Ahmed et al. [15] tried to translate this value to a log-uniform distribution from 2.56 to 7.67 as noticed during the periods of heaviest shedding among mild cases of COVID-19. These results provide further support to estimate the number of infected cases within the community via wastewater-based epidemiology, as a new paradigm in public-health assessment. In anal swabs of hospitalized cases of coronavirus disease, viral loads of 10^5^ gc/swab for SARS-CoV-2 has also been reported [176].

Despite the extensive reports regarding fecal shedding of SARS-CoV-2 RNA from COVID-19 patients, none of the reported studies to date have provided information on the viability of infectious virions, capable of initiating disease in a susceptible individual, in stool samples possibly. This could partly be assigned to the difficulties in maintaining human viruses in vitro as well as the requirements for trained staff and specialized equipment. Nevertheless, the literature on this topic has only just started to increase. These studies typically use human or animal cell lines (e.g., Vero E6 cells, based on monkey kidney cell lines) to analyze qPCR positive samples through cell culture infectivity assay, integrated cell culture-qPCR (ICC-qPCR), and EMA/PMA-RT-qPCR which are then followed by genotyping [177]. Two studies found cultivable SARS-CoV-2 in fecal samples from hospitalized patients, reinforcing the scientific speculations on the serious consequences for transmission through exposure to feces [34,66]. Although Wang et al. [34] pointed toward detection of viable SARS-CoV-2 in stool samples, it is worth noting here that no evidence of viability was provided. However, in contrast to these findings, a more recent study has been unable to demonstrate the isolation of culturable virus from feces despite high viral RNA copies [64]. Although firm evidence is lacking, there are few case reports of SARS-CoV-2 shedding in urine [178,179,180]. There does, nevertheless, appear to be very little evidence to directly support such studies which thus remain largely speculative at this stage (which could be attributed to the plausible secondary contamination) until further research has been done.

In closing, although the literature on the occurrence and survival of virus in feces shows heterogeneous results, fecal–oral transmission of SARS-CoV-2 requires comprehensive and more nuanced interpretation. One of the issues that emerges from clearance of live SARS-CoV-2 virus or even viral RNA by feces of affected patients is potential enteric transmission to health professionals and those working with human excreta in the hospitals whose rate of infection is extremely high. The plausible effect of public toilets in the spread of disease, which may be places at risk of infection, particularly for patients having gastrointestinal symptoms, such as diarrhea, also remains purely speculative. In this essay, local authorities are recommended to consider disinfectant addition as the preferred emergency sanitation treatment and try to put forward the relative merits of the 0.5% or 2% chlorine solutions to disinfect human excreta, as has been suggested during the Ebola outbreak [181].

### 3.2. Occurrence of SARS-CoV-2 in Wastewater

Both viable SARS-CoV-2 and nonviable virus (and associate viral debris such as RNA fragments, mRNA, or capsid subunits) may found their way into sewage trough bodily excreta, including hand washing, saliva, sputum, vomit, feces, and possibly urine which are subsequently disposed of in wastewater. During the last seven months, considerable efforts have been devoted to detect SARS-Coronavirus-2 in sewage in several countries, particularly in high- and upper-middle-income communities. Details of these reports are summarized in Table 2. The imbalance between the number of studies in developed countries and those on the broad spectrum of under-developed and resource-limited communities clearly indicates that much work has yet to be accomplished. Gathering such comparative data would be the best way to ensure adoption of WBE worldwide for monitoring pandemics—not just for controlling Covid-19, but also for future epidemics.

Most of the studies on virus surveillance in wastewater have focused on the presence of viral fragments of SARS-CoV-2 RNA and little has been documented on viral titers or the potential infection risk in wastewater. The presence of SARS-CoV-2 in wastewater was first described by Medema et al. [17] in the Netherlands, six days before the first cases were officially announced. Nevertheless, Mallapaty et al. [182] provided a shred of evidence to support the presence of virus in wastewater at Amersfoort, the Netherlands, days before the first cases were reported. In parallel, Lodder and de Roda Husman [112] pointed towards the first report of SARS-CoV-2 detection at Amsterdam Airport Schiphol, the Netherlands, via human wastewater surveillance. They worked on 24-h 10 L composite samples exploiting quantitative RT-PCR methodology and provided the first evidence of virus presence in the wastewater four days after the first cases of COVID-19 were identified in the Netherlands [112]. This evidence was corroborated by two other studies in untreated wastewater in Italy [126] and Turkey [107]. The occurrence of SARS-CoV-2 RNA in the wastewater and sewage sludge was also reported even in a low-prevalence area in Spain [109], while another study reported the presence of corresponding virus in raw and partially-treated sewage in New York, USA [183].

Wu et al. [110], through monitoring a wastewater treatment plant in Massachusetts, USA, reported the concentration of SARS-CoV-2 in wastewater samples as ~10^4^ copies/100 mL using a polyethylene glycol (PEG 8000) concentration method and CDC N1, N2 and N3 assays as has been used by Medema et al. [17]. One of the first reports of SARS-CoV-2 detection in public sewage was achieved in Brisbane, Australia [15]. This study reported on the positive detection of viral genome in untreated wastewater during COVID-19 epidemic peak. The study applied RT-qPCR to obtain quantification cycle (C_q_) values and was followed up with WBE approach to estimate the prevalence of affected people in the catchment. The viral titers were estimated as 12 and 1.9 gc/100 mL of untreated wastewater based on the obtained C_q_ values in two studied samples (37.5 and 39, respectively). Although these C_q_ values are comparable to those that the virus has in the feces, there is a clear separation from clinical pharyngeal swab specimens [35]. This could be either attributed to the lower rate of viral shedding in stool or problems with the way the original data was collected (as discussed in detail in Section 3.1).

To summarize, infectious SARS-CoV-2 has not been detected in untreated or treated sewage [184]. However, RNA components of SARS-CoV-2 have been detected in untreated sewage and sludge in a number of countries and municipalities, with RNA signals, generally starting around the same time cases were first reported (February and March 2020) and increasing as the number of confirmed cases increased. Detection of viral genetic materials (RNA) alone, however, does not necessarily confirm the presence of viable infectious virus or a risk of infection. In general, RNA fragments are much more persistent in the sewage matrices than infectious virus, and there is no clear correlation between RNA load in sewage samples and infectiousness. However, tracing SARS-CoV-2 RNA ‘signature’ is at least a ‘smoking gun’, allowing local authorities to judge the possibility of infection hazard. We cannot, of course, exclude that exposure levels could be related to yet unrecognized or unmeasured confounders. Quantifying transmission risk, indeed, necessitates knowledge of the minimum infectious dose of the virus, which for SARS-CoV-2 has not been yet determined, as well as data on the amount of virus that individuals are exposed, too. An analysis of the data available in the literature already confirms the occurrence of SARS-CoV-2 RNA in untreated wastewater with maximum concentrations over 10^6^ copies per liter. To further expand the investigations of the occurrence of SARS-CoV-2 RNA in sewage systems, several studies dealing with viral titers in treated effluent were retrieved (Table 2). Three of those reports specifically addressing detection of SARS-CoV-2 RNA in effluent announced viral loads up to 10^4^ gc/100 mL [185]. The study in Paris detected the SARS-CoV-2 RNA in treated wastewater as well, with concentrations of up to approximately 10^5^ copies per liter [106], forcing the scientific speculations on the serious consequences for the sake of public health.

Thus far, published data on the presence of SARS-CoV-2 in wastewater mainly come from municipal sewage, with limited data on virus presence in hospital effluents. SARS-CoV-2 is enveloped and thus less stable in the environment compared to non-enveloped human enteric viruses with known waterborne transmission (such as adenoviruses, norovirus, rotavirus, and hepatitis A virus) In support of these findings, viral fragments of SARS-CoV RNA were traced in 100% of untreated wastewater and 30% of disinfected effluent samples collected from a Chinese hospital during the SARS outbreak in 2004 [186].

### 3.3. Detection and Quantification of SARS-CoV-2 in Wastewater

**Figure 4:**
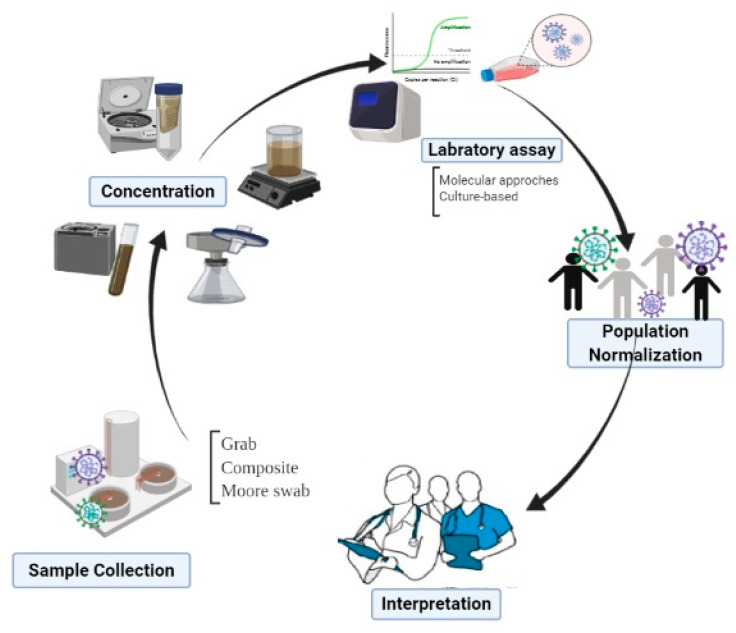
Steps in detecting and enumerating of SARS-CoV-2 in sewage.

Detecting and enumerating of SARS-CoV-2 in the sewage requires a number of steps (Figure 4), each undoubtedly facing a number of challenges, many of which are often shared by existing reports [15,17,110]. Some of these challenges seriously affect the resultant outcome, as we will discuss in the following sections along with techniques developed to overcome them.

#### 3.3.1. Challenges Involved in Obtaining Representative Samples

Given the heterogeneous nature of wastewater, this type of research is fraught with a statistically representative sampling of sewage. It is just as important to take a representative sample as it is to analyze the sample correctly. The question of what constitutes a representative sample has been addressed previously [120,187]. The limits of data variability are potentially influenced by many parameters, including the sampling procedure. Perhaps the greatest question remains whether the grab or composite sampling method should be performed. The grab sampling approach is likely to be utilized when either the level of viruses or the content of solids is relatively high, as is the case for raw wastewater. A composite sample, on the other hand, is a sample unit that is created by combining or pooling multiple sample units. Thus, if the study objective is to get a good estimate of the average concentration of infectious agents in a large volume of environmental matrix—like public raw sewage—individual replicates from a sampling point could be physically combined to create one sample unit that is sent for analysis. The advantage of this method is that it obtains values that are expected to more closely follow a normal distribution and hence meets the assumptions of standard statistical tests. The appropriate use of composite samples can indeed be a cost-effective way of producing precise and accurate estimates of average levels, among others [188]. Thus, the challenge is to effectively sample and encompass the variability in viral RNA, particularly in quantitative studies set out with the aim of sewage surveillance to estimate the infected people through the accurate detection and quantification of viral load by wastewater-based epidemiology. Except for individual variability in viral shedding coupled with viral persistence in the sewer network, this approach encounters huge challenges concerning variations in wastewater flow rates. Although it might not be a serious issue in large sewer systems, higher domestic water usage at peak times (e.g., mornings and evenings) [189] could result in reduced concentrations of viral load at these times because of dilution effects.

It is further important to bear in mind the possible bias in viral loads due to regional rainy days and melting snow again with a clear result of sewage dilution in combined sewers. Daily mean temperature and amount of rain therefore not only may affect the number of positive SARS-CoV-2 cases in the specific catchment area, but also gene copy numbers. In accordance with these hypothesis, Wurtz et al. pointed to two episodes of rain (September 19 to 22 and November 7 to 8) in their sewage monitoring for SARS-CoV-2. Surprisingly, they opined that the quantity of viruses did not fall during the first episode, but possibly with the second [190].

**Figure 5:**
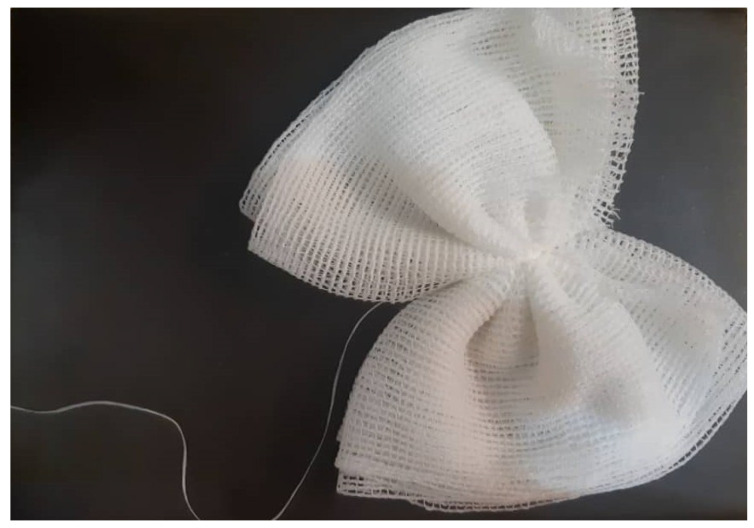
The Moore swab [192].

Retention time in the sewer network (i.e., the time a sample takes to travel in the sewer pipe) indeed may have a profound effect on the structure of the sample. Thus, monitoring across the network may assert the merit of priority or even better representation of the situation. A composite sampling approach, and not just one snapshot in time, thus will help to alleviate the problem and to obtain near real-time concentration of viruses detected in wastewater. Dealing with the limitations such as intermittent shedding of enteric pathogens by humans and the effects of dilution, Moore swab as a classic environmental surveillance tool, whereby a gauze pad is suspended in flowing wastewater to trap microorganisms (Figure 5), may regain appreciation in sewage fingerprinting [191]. The method is advantaged by its simplicity and affordability and is well suited to resource-limited communities. The use of Moore’s technique has been re-surfaced in recent conference proceedings describing environmental surveillance programs for enteric pathogens, such as Salmonella and Vibrio cholera [191]. Most recently, Rafiee et al. reported that the use of Moore swabs outperforms composite and grab sampling methods for SARS-CoV-2 monitoring in wastewater, providing promising results as to be the best sampling protocol to adopt when planning a sewage monitoring campaign particularly under WBE [192].

The sampling process is compounded by the virus’s degradation in a relatively short space of time. Thus, sample transport and storage need to be carefully controlled—usually keeping in a refrigerated container (or icebox) at a temperature ˂10 °C. The elapsed time allowed between sample collection and analysis ≤24 h. If sample analysis within the 24-h time limit is not possible, long-term storage of samples should be operated at −80 °C. Viruses are also in the throes of inactivation by the presence of chemicals, e.g., detergents and cleaning chemicals which are used copiously during a pandemic.

#### 3.3.2. Challenges Involved in Concentration/Enrichment of SARS-CoV-2 in Wastewater

Given the potential low concentrations of virus titer in environmental matrices, sensitive detection of viral particles in such matrices like untreated wastewater and sewage effluent necessarily entails samples to be concentrated before they can be detected. In Nucleic-Acid-Based methods, the process may include a stage to physically concentrate the infectious particles (e.g., through filtration or precipitation), and an amplification stage (repeated replication of RNA to allow its detection). Wastewater samples are often centrifuged or filtered to eliminate debris. The process is followed by enrichment of viruses contained in sewage samples; the best available approaches at present include electronegative membrane filtration (0.45 µm) [15], skimmed milk flocculation [193], ultrafiltration [111,194,195] or polyethylene glycol precipitation [195,196], aluminum flocculation [109,197], or ultracentrifugation [106,113] enabling, as reported, 20 to 800 folds concentration. Meanwhile, except for Medema et al. [17] and Randazzo et al. [109] suggesting 3–50% viral recoveries, the literature is limited with respect to the percentage recovery of SARS-CoV-2 from sewage samples. This could be possibly due to the risks associated with handling the virus and requirements for a high-containment facility in order to fulfil biosafety guidelines. SARS-CoV-2 is classified as a hazard category 3 pathogen, which means that any work with the viable virus necessitates the use of biosafety level 3 (BSL-3) laboratories. Detection of RNA, however, could be operated in BSL-2 diagnostic laboratories which are more widely available and cost less to access. The CDC have recently released guidelines for testing environmental specimens to monitor SARS-CoV-2 in wastewater samples, clearly declaring that “Procedures that concentrate viruses, such as precipitation or membrane filtration, can be performed in a BSL-2 laboratory with unidirectional airflow and BSL-3 precautions” [198]. This same general result was concluded by Haramoto et al. [199] and Ye et al. [200], who pointed towards the inefficiency of standard virus concentration methods to recover enveloped viruses (e.g., SARS-CoV-2) from environmental matrices. The results thus remain highly controversial concerning reproducibility within and between studies. The use of appropriate process controls (e.g., spiking the sample with surrogate animal viruses of the same family or genus—such as heat-inactivated SARS-CoV-2 provided by ATCC or Pepper mild mottle virus (PMMoV)—having similar structure to the target pathogen before concentration) has been proposed by Randazzo et al. [109] and Farkas et al. [201] to estimate viral recoveries. The volume of sewage to be concentrated is another important parameter that still largely remains unknown but has a substantial impact on the results, which is discussed in Section 3.3.3 in more detail.

The tendency of viruses to biosolids paves the way for the partitioning of SARS-CoV-2 into sludge, particularly in a primary sedimentation tank because of its longer retention time. Except for a recent study conducted by Garham et al. [150], this issue has not been adequately addressed in the literature. Accordingly, monitoring primary sludge in a WWTP increases the chance of higher detection signals, as has been pointed out by Peccia et al. [202], who reported that the concentrations of SARS-CoV-2 RNA ranged from two to three orders of magnitude greater than the corresponding values previously reported in untreated wastewater. Nevertheless, it is important to note that such samples may inherently impose more inhibition in downstream analyses.

#### 3.3.3. Challenges Involved with Laboratory Assays Dealing with SARS-CoV-2 in Wastewater

The resultant concentrated sample materials are then analyzed for the presence/enumeration of SARS CoV-2. This can follow two broad approaches targeting either (i) virus functional or structural motifs (e.g., RNA fragments, antigens, or other associated viral debris) or (ii) intact infectious virus particles [20].

#### 3.3.4. Nucleic-Acid-Based Methods

Molecular approaches do not substitute for culture-based methods and address virus nucleic acids, which in SARS-CoV-2 is viral RNA. The efficiency and representativeness of genetic material—viral RNA—recovery from raw wastewater or treated effluent samples are fundamental concerns in molecular techniques. Today, RNA extraction is by and large carried out using commercial kits supplied by a variety of manufactures. The CDC have also recently published a report on Real Time RT-PCR Diagnostic Panel which deals with a list of RNA extraction kits that can be used for SARS-CoV-2 [198].

In addition to the limitations touched upon above regarding virus concentration/enrichment, the literature is somewhat contradictory with respect to the performance of PCR-based methods (PCR, RT-qPCR or allied approaches) designed for the detection of SARS-CoV-2 in sewage samples [15,17,109,110]. A specific segment of the viral RNA is targeted in these methods, paving the way for rapid, sensitive, and accurate strain-level detection of target(s) in real time RT-PCR assays [203]. Degradation products (such as RNA fragments) from the virus could also be selected as targets. As a quantitative nuclear-derived method, RT-PCR monitors the amplification of a targeted part of viral RNA using reverse transcriptase (to generate a DNA strand complementary to the RNA) and polymerase (to replicate the strand repeatedly) enzymes as far as it can be traced by fluorimetry [17]. This measurement is expressed as cycle threshold (Ct), a relative value that represents the cycle number (repeated PCR reactions) at which the amount of amplified DNA reaches the threshold level. Ct values can be alternatively normalized and expressed as gene copies (gc) per unit of volume (e.g., mL or L) or mass (e.g., g or Kg). Several questions remain unanswered at present. Accordingly, capability of detection and quantification are important performance characteristics of RT-PCR, as with any analytical technique. There has been considerable attention paid in recent works to unravel SARS-CoV-2 Detection and Quantitation Limits (LOD and LOQ) in wastewater, whereby early estimates put forward on LODs of a few thousand copies per liter with LOQs as reasonably 10–20,000 gc/L [204]. As of relative merits to cerebrate, an infected COVID-19 patient excrete approximately 10^8^ to 10^12^ viral particles per day (see Section 3.1). However, more research on this topic needs to be undertaken. The ‘N1′ to ‘N3′ primer/probe sets each targeting a different region of the nucleocapsid (N) gene have been suggested by US CDC and their specificity against other viruses, including human coronaviruses, has been reported [205]. Corman et al. [206] in the Netherlands worked with a set against the RNA-dependent RNA polymerase [RdRp] gene, envelope [E] gene, and nucleocapsid [N] gene for detecting beta coronaviruses, including 2019-nCoV. Very recently, amplification of viral RNA targeting the ‘ORF1ab’ gene has also been reported in some studies concerning sewage surveillance to estimate the infection incidence within the population [75,119,207].

Although firm evidence is lacking, a combination of SARS-CoV-2 molecular tracing experiments in sewage samples put substantial differences for amplification of the viral RNA with different primer/probes. For instance, compared with the results for the sensitivity of primer/probe sets as ‘N1’ = ‘N3’ > ‘N2’ on SARS-CoV-2 RNA in clinical samples reported by US FDA, Medema et al. [17] observed a higher frequency of positive amplification targeting ‘N1’ and ‘N3’ genes, while ‘N2’ assay did not detect SARS-CoV-2 in wastewater samples. Moreover, ‘N1’ outperformed ‘N3’ and ‘E’ primers. In support of these findings, Rimoldi et al. [119] reported a distinct advantage of ‘ORF1ab’ gene over the ‘N’ and ‘E’ genes, which could be attributed to primer/probe sensitivity. To obtain a broader picture, Medema et al. [17] reported a positive signal for the ‘N2’ primer/probe set looking for a RT-qPCR signal between 40 and 45 cycles. It indicates that the role played by natural inhibitors in the gene amplification reaction of viruses might be solely responsible for increasing the Ct values, causing false-negative results [176]. It is expected that these findings will have important implications for using multiple primer/probe sets for future sewage surveillance projects.

Problems with the presence of inhibitory organic co-contaminants (e.g., humic and extracellular polymeric substances), which quite often afflict with the reverse transcription and polymerase enzymes, thus may invalidate the findings. Unfortunately, most concentration/enrichment methods could worsen the problem through the simultaneous concentrating and extracting organic compounds together with the targets [201]. Inhibition of RT-PCR reactions was reported by Medema et al. [17] in their initial trials. They stated that they were able to overcome these difficulties by the addition of BSA (Bovine Serum Albumin) to the reaction mixture. To the best of our knowledge, the report about assistance of another chemical or even process (which could be adsorption, ion exchange, etc.) to deeply remove inhibitory organic/inorganic compounds from sewage samples before PCR test targeting SARS-CoV-2 remains scarce. The use of polyethylene glycol (PEG) [110] and RNA magnetic beads [146,208], among others, has been previously reported to help get rid of inhibitors, which could be followed in downstream RNA targeting PCR. Addressing these problems calls out for a tremendous amount of research to be conducted to develop new robust methods, while at the same time concentrating the PCR targets. The Clustered Regularly Interspaced Short Palindromic Repeats (CRISPR) based on Cas proteins [11,12] in combination with isothermal amplification and lateral flow assay has shown a promising potential in the detection of SARS-CoV-2 [209]. The isothermal PCR and visual observation make a faster alternative to the US CDC and SARS-CoV-2 real-time RT–PCR assay, with 95% positive and 100% negative predictive agreements, respectively [210].

#### 3.3.5. Culture-Based Methods

It is important to bear in mind that the presence of viral RNA does not indicate the infectivity state of the target virus or a risk of infection. The virus genetic materials (including RNA fragments) are much more stable in the sewage than infectious virions. While, it can be inferred, with due care, that high counts of RNA provide an early sign of a potential hazard, there is no clear correlation between RNA concentration and infectiousness. With respect to its occurrence in sewage, viable SARS-CoV-2 probably enters mainly from shedding in the stool and to lesser extent from saliva and sputum. To assess whether there is a potential infection risk, sewage samples ideally need to show that the virus is infectious (viable). An assessment is further compounded by the lack of dose-response data, which means that it needs to come with details of whether the virus is present in adequate quantity in the particular location that has been monitored or sampled for transmission to take place. As of the time of writing, one of the reported studies was designed to detect live virus in sewage or deal with the potential infection hazard. The paucity of data on virus survival may have something to do with viable SARS-CoV-2 with regard to biosafety prerequisites (BSL-3 containment facilities, as mentioned previously) as well as the virus half-life in wastewater that seems to be very short [170]. Preliminary estimates of SARS-CoV-2 persistence in the water environment, as a metric for survivability and fate of SARS CoV-2 in wastewater matrices either municipal or hospital, are only a few days in the absence of disinfection measures [211]. However, some studies draw a different conclusion, suggesting that infectious viral titers may be able to survive longer than presumed [212,213,214]. It is, however, worth noting that the experimental conditions in which virus survivability has been examined coupled with the longer survival of the viral surrogates exploited in those studies seem to disprove this idea. According to a recent published report by Rimoldi et al. [119], SARS-CoV-2 remained infective on the time scale of a few hours. To obtain a clearer picture, they were unable to detect viable virus in wastewater samples just after 6–8 h from excretion of the viral particles in feces to the sewage sampling point. Indeed, the infectivity of virus is commonly confirmed in human or animal cell lines which not only is time-consuming (4–5 days for results) and resource-intensive, but also requires finding the appropriate host, as discussed before (Section 3.1). Another further concern in this regard is distinct susceptibility of cell lines exploited in culture-based assays which may not be infected as readily as respiratory tract cells which are the main targets of SARS-CoV-2 infection. This could be assigned to the plausible differences in the number of ACE2 receptors, the binding site for SARS-CoV-2.

As a result, it was not possible to draw a meaningful conclusion in relation to the sewage networks and role of WWTPs in the transmission of SARS-CoV-2. Nonetheless, the presence of enveloped viruses like coronaviruses, which undergo rapid inactivation outside the human host, have also been previously reported in wastewater [215]. Due to the lack of adequate evidence for the survival of SARS-CoV-2 in sewage, the infectious capacity of virus in sewer networks and wastewater treatment plants still needs more research that accounts for both viable and non-viable particles. It is worth bearing in mind that even if the presence of infectious virus is confirmed through culture-based techniques, it does not necessarily mean that it is present at a sufficiently high dose to pose a biohazard.

Interestingly, it was reported that the viral pathobiology still continues to be an evolving aspect that potentially shows virus genome is undergoing recurrent and independent mutations. The later demonstrates ongoing adaptation of SARS-CoV-2 to its novel human host [216]. The virus indeed is possibly in the throes of eventual mutations in the environmental compartments. Currently, the emergence of genomic diversity and mutations in virus come from symptomatic SARS-CoV-2 infected cases, while there are larger asymptomatic populations. Monitoring the mutations and evolution of virus in the sewage systems thus may provide a broad and accurate picture of viral mutation rates in the community level within the study area. Such information not only has the potential to inform on targets for drugs and vaccines, as has been previously highlighted, but also may subsequently assist in post-vaccination campaigns in which the virus mutation rate becomes a crucial factor in combating the pandemic.

### 3.4. Potential Risks for Wastewater Operators and Workers in the Vicinity of the Virus and the Environment

In general, raw wastewater contains, at any time, several pathogens and therefore poses certain health risks to whoever may come into direct contact with wastewater or be exposed to aerosolized droplets containing pathogenic virus, particularly near wastewater pumping stations and mechanical agitation. This is particularly important in less developed countries, where occupational exposure in WWTPs may warrant additional concern since the protocols of personal and collective protective equipment use is not as stringent as it is in the developed communities. Therefore, all such environments should have in place adequate health-safety procedures. The viral loads of SARS-CoV-2 in sewage at the entrance of the WWTPs are estimated to be 1.03 × 10^2^ to 1.31 × 10^4^ gc/mL (0.1 to 13.06 PFU/mL, respectively) [14]. Although detection of viral RNA in sewage does not necessarily mean that the sampled location contains infectious virus, the estimated risk of infection for workers based on quantitative microbiological risk assessment (QMRA), was less than WHO’s benchmark value of 10^−3^ for the moderate scenario, which has been highly advisable for workers in WWTPs. Nevertheless, given the lack of adequate evidence for the transmission of COVID-19 due to exposure to wastewater, WHO indicates that sewage has not yet played any role in the current pandemic [5]. In addition, evidence to date has demonstrated that the major infectious properties are destroyed during the wastewater treatment processes and the exposure to the virus is thus considered to be negligible compared to direct contacts between humans [184].

Finally, the potential ecological risks to the wider environment are also of great concern following poorly treated and untreated wastewater disposal or land-use of the raw/treated sludge. Some recent reports stressed that SARS-CoV-2 might have the ability to infect semiaquatic secondary animal vectors as well as several mammalian species [9,217,218,219]. Species close to outlets are in the throes of coming into direct contact with infectious virions from which it would likely become endemic in the secondary host, facilitating secondary transmission risk of virus. In addition, significant signals of selection and accelerated evolution in the ACE2 coding sequence across all mammals were found, and specific to the bat lineage [219]. Nevertheless, more studies are needed to address this concern properly.

### 3.5. Population Size Normalization

One of the greatest challenges in wastewater surveillance applications (e.g., WBE) for public-health studies is the accurate size estimation of contributing population to infer infection rates in a sewershed catchment. Uncertainty in connecting the observed viral signal in raw sewage to the contributing population could lead to a false-positive or -negative disease prevalence estimation in the community and can undermine WBE as a monitoring tool in situations such as post-vaccination. In this regard, both biomarker and census methods can be used, which should be independently collected and integrated with common units. Biomarkers are stable and human-related chemical or biological compounds whose quantity and quality can implicitly help with population estimation. The quantity of biomarkers would provide the basis for measured viral concentration and ensure that differences in viral loads could not be ascribed to population changes. Provided that observed viral concentrations are significantly high relative to the estimated population, a viral outbreak will be portended. Chemical biomarkers such as creatinine, cholesterol, coprostanol, nicotine, cortisol, androstenedione, ammonium, and the serotonin metabolite 5-hydroxyindoleacetic acid (5-HIAA) have been proposed as an endogenous or exogenous human substance to estimate real-time human population size from sewage samples [220]. Human mitochondrial DNA, human RNA, or CrAssphage (a benign virus that is common in the human gut) can be used in the same way [85,201]. Human nucleic acid has a great potential to act as a population biomarker due to its limited affinity to other species in wastewater, stability, constant excretion by humans, and the possibility of being quantifiable using the same pipelines and platforms as the viral nucleic acid of interest. For instance, Ling et al. [221] developed a new method to normalize data generated from sewage in view of recent developments in species-abundance distributions of microbial ecosystems. In their non-parametric model (Microbiome Census) metagenomic taxon-abundance of gut associated microbiome exhibited a good estimation of human population size in small communities.

Loading-based population proxies such as the copy numbers of crAssphage, pepper mild mosaic virus, and adenovirus are better than other human pathogenic viruses such as norovirus, due to demonstrating a strong seasonality in wastewater. Nevertheless, the lack of consensus among experts for accurate population estimation methods still remains as a major hurdle in adapting WBE for SARS-CoV2, and more research needs to be done on this front.

#### 3.5.1. How Practical Is the Quantification of Virus in Wastewater Surveillance?

As discussed in previous sections, the limits of data variability are potentially influenced by sampling procedures (time, volume, and frequency), concentration/enrichment, detection, and quantification of virus in wastewater, as well as positive controls among others, so that detected amounts of RNA should be cautiously compared among studies. Thus, to obtain accurate results with statistical significance, applying standardized protocols is a must-have for sewage surveillance to ensure reproducibility and comparability of outcomes.

The joint research center of the European Union (JRC) has shown a correlation between the number of people infected in a sewershed and the viral load in wastewater [222]. Results also showed that the viral load in the sewage increases before the number of infected persons increases [22,223]. Therefore, the number of people infected in an area could be approximately estimated based on quantities of virus found in wastewater. However, it should be noted that each country might have a different testing procedure. In addition, while wastewater surveillance can reveal the presence of virus, it cannot tell whether this is from residents or from commuters (or tourists). Therefore, the wastewater surveillance estimates should be cross-checked by other sources of information to draw a more comprehensive conclusion.

#### 3.5.2. How to Use and Interpret the Wastewater Surveillance?

Despite the challenges of wastewater surveillance for SARS-CoV2 detection in a community that have been mentioned before, it can still provide very useful information. However, it should be mentioned that this information should always be used as a complement to other epidemiological data when available. In the case of a lack of such reliable epidemiological data, wastewater surveillance can still be used in less favored conditions. Nevertheless, wastewater surveillance can be useful in three main categories [222,223]: As an early-warning tool to detect (re-)emergence of the pandemic in a specific sewage catchment area; As a management tool to identify the prevalence and trend of infection, as well as to determine low- or high-risk areas, for example, if surveillance data shows the absence of virus in wastewater, the corresponding community can be considered as of low risk; and As a safety factor in cases where testing of residents shows negative results but the virus is detected in wastewater, then further investigations should be prescribed—it can also be useful to evaluate the safety of tourists’ facilities with a controlled environment, such as cruise ships and other similar facilities.

In addition, wastewater surveillance has the potential for use as a tool for guiding and monitoring vaccination efforts [223,224]. To measure vaccine efficacy in real-world settings, there is a reliance on ongoing transmission of SARS-CoV-2, which is contradictory to public-health promotion efforts [225]. In contrast, the level of ongoing community transmission can be monitored by wastewater surveillance, which can provide insights into the impact of the vaccination programs. There are currently limited data on the efficacy of the vaccines to protect against different COVID-19 variants. Wastewater monitoring may provide evidence on the SARS-CoV-2 RNA viral load and viral shedding trends during the roll-out of vaccinations in communities. Furthermore, wastewater surveillance can provide an early-warning system for prioritizing hotspots where vaccination coverage is most urgently needed. Finally, targeted and untargeted sequencing of wastewater pathogens has the potential to track the spread of new specific sequence variants and identify mutations for which vaccinations may be needed to be modified [201,226].

## 4. Knowledge Gaps and Concluding Remarks

A comprehensive literature review was done to investigate the current state of knowledge and to identify the research gaps regarding the occurrence of SARS-CoV-2 in the feces of affected individuals and its dissemination in wastewater. In general, the current literature already confirms the occurrence of SARS-CoV-2 RNA in untreated wastewater with maximum concentrations over 10^6^ copies per liter. While broad consensus has yet to form on the degree of risk, it is increasingly acknowledged that the presence of even SARS-CoV-2 genetic materials is of concern in both the raw and treated effluent and its potential to cause viral dissemination in workers in wastewater-treatment plants and in biota is clearly an issue which should inform environmental policy. Concern has also been raised about vegetables irrigated with untreated or partially treated wastewater, particularly in under-developed and developing countries. The lack of sewage separation (urban runoff from domestic effluents), which brings about combined sewer overflows in the events of heavy rainfalls, could also be a rationale for the occurrence of viral loads in water bodies [226]. Nevertheless, it should be recognized that there are still substantial knowledge gaps in the ever growing concern of potential infection hazard. An important aspect relating to the infection risk is that such samples ideally need to confirm that the virus is viable (infectious) and also demonstrate an adequate quantity for transmission to take place. Whilst this is a burgeoning area of research it needs to be made if individuals are likely in the throes of an infectious dose in the particular location where the sample was taken (e.g., toilet environments, sewer works, and WWTPs). These open questions warrant ad hoc investigations.

Based on the best available evidence at the present time, standardization of virus analysis in sewage is still needed. Areas that require the most improvements are sampling procedures, concentration/enrichment, detection, and quantification of virus in wastewater, as well as positive controls. Considering the quality of individual studies, problems with the protocols through which the original data was collected may invalidate these findings. Standardized protocols will indeed allow reproducibility and comparability of outcomes and would be reflected in the quality of data that are needed in policy and management interests. Testing in representative samples could also greatly attenuate the sources of uncertainty in model simulations, particularly in projects setting out to estimate numbers of infected people through wastewater-based epidemiology. Given the aforementioned differences in targeted procedures, conclusions on loading comparisons among studies and communities are difficult to draw. The presence of inhibitory organic co-contaminants in wastewater, which sometimes interferes with the PCR analysis, may invalidate the results. More research is thus needed regarding the assistance of other chemicals or processes (e.g., adsorption, ion exchange, etc.) to effectively remove inhibitory compounds from sewage samples before PCR test targeting SARS-CoV-2.

Another important knowledge gap to consider stems from the fact that the overwhelming majority of literature has focused on molecular methods to detect SARS-CoV-2. A confounding issue, however, is the infectious capacity of virus in feces and sewage systems since almost all studies just focused on viral genome detection/quantification. Nevertheless, given the lack of adequate evidence for the transmission of COVID-19 due to exposure to wastewater, it has been reported that sewage has not yet played any role in the current pandemic. In addition, evidence to date has demonstrated that the major infectious properties are destroyed in sewers and during wastewater treatment processes. The exposure to the virus is thus considered to be negligible compared to direct contacts between humans. A key research need is to investigate the correlation between PCR signal and infectious virus concentrations.

The nature of interactions between the virus in raw wastewater and other species (vertebrates and invertebrates) is yet to be fully elucidated since there are reports on secondary transmission of virus in animal vectors and several mammals. While by means of improved research techniques and announced progress in vaccine development, there is hope that the pace of disease will begin to slow, and studies looking into monitoring the traces of virus mutations in sewage as a broad picture of viral mutation rates in the community level within the study area particularly in post-vaccination campaigns should be encouraged. Genetic sequencing of virus in public sewage samples may help to explore the prevalence of different strains circulating within a specific catchment area, giving an indication of the evolution and spread of the disease. It is expected that the results of such research will have important implications for the sake of public health.

Results further showed that selecting the most accurate population estimation method for wastewater-based epidemiology studies is still a challenge. While census and biomarker studies have shown promising results, more research is needed to develop the best population size normalization method with respect to SARS-CoV-2. Although the number of people infected in an area could be approximately estimated based on quantities of virus found in the wastewaters, these estimates should be cross-checked by other sources of information to draw a more comprehensive conclusion. Furthermore, wastewater surveillance can be useful as an early-warning tool, a management tool, and/or a safety factor. Finally, wastewater surveillance has the potential to evaluate the effectiveness of vaccination programs, track the spread of specific sequence variants of SARS-CoV-2 and identify mutations for which vaccinations may be needed to be modified.

## Figures and Tables

**Table 1:** Selected data extraction fields employed in the literature review protocol.

Study	Country	Positive Stool Samples/Total (%)	Diarrhea	Duration of SARS-CoV-2 RNA Positive (Day)	Duration of Persistent Positive Faecal RT PCR after Negative NP RT-PCR (Day)	Same Pathogens Found in Urine	Viral Load	Ct
TAN Xin et al. [21]	China	10/13 (77)	NA	NA	12	NA	NA	NA
Cai Jiehao et al. [22]	China	5/6 (83)	0/6	NA	18–30	No	NA	NA
Ziying Lei et al. [23]	China	4/7 (57.1)	5/20 (25)	4–16	5-6	NA	NA	NA
Jasper Fuk-Woo Chan et al. [24]	China	0/7	NA	NA	NA	0/7	NA	NA
Yajun Yuan et al. [25]	China	6/6 (100)	NA	0-28	NA	NA	NA	NA
Stephanie A. Kujawski et al. [26]	United States	7/10 (70)	3/10 (30), all had viral RNA detected in stool	NA	NA	No	NA	24.1–39.4
Francois-Xavier Lescure et al. [27]	France	2/5 (40)	NA	NA	10	No	6·8–8.1 log10 copies	NA
Wei Liu et al. [28]	China	0/19 (0)	NA	NA	NA	No	NA	NA
Chunbao Xie et al. [29]	China	8/9 (88)	0/9	NA	NA	No	NA	NA
Lijuan Chen et al. [30]	China	1/1 (100)	NA	NA	NA	NA	NA	NA
Tongqiang Zhang et al. [31]	China	3/3 (100)	NA	10–13	NA	NA	NA	NA
An Tang et al. [32]	China	1/1 (100)	NA	17	10	NA	NA	ORF1ab: 32.6; Youjiang nucleoprotein gene: 33.7
Juan Liu et al. [33]	China	4/4 (100)	NA	8–11	NA	NA	NA	31.7–36.6
Wenling Wang et al. [34]	China	44/153 (29)	NA	NA	NA	0	NA	31.4
Xiao-Shan Wei et al. [35]	China	28/84 (33)	26/84 (31)	NA	NA	NA	NA	NA
Chaoqun Han et al. [36]	China	12/22 (54.5) respiratory only: 3/5 (60), digestive + respiratory: 1/7 (14.3), and digestive only 8/10 (80)	117/206 (56)	NA	NA	NA	NA	NA
Xiang Ma et al. [37]	China	8/27 (29.6)	NA	28	NA	NA	NA	NA
Yuhan Xing et al. [38]	China	3/3 (100)	NA	28	8	NA	NA	NA
Yu-pin Tan et al. [39]	China	3/10 (30)	NA	6–10	11	NA	NA	NA
Barnaby Edward Young et al. [13]	China	4/8 (50)	3/17 (17)	NA	NA	0/10	NA	29–36
Iek Long Lo et al. [40]	China	10/10 (100)	8/10 (80)	2–17	9-11	0/10	NA	NA
Fei Xiao et al. [41]	China	39/73 (53.49)	26/39 (66)	1–12	NA	NA	NA	NA
Xiaowen Hu et al. [42]	China	0/59	NA	NA	NA	0/59	NA	NA
Maria Effenberger et al. [43]	Austria	12/40 (30)	22/40 (55)	NA	NA	NA	NA	NA
JingCheng Zhang et al. [44]	China	5/14 (35.7)	0/14	1–3	1	NA	NA	NA
Michelle L. Holshue et al. [45]	United States	1/1 (100)	1/1 (100)	NA	NA	NA	NA	36–38
Lu Lin et al. [46]	China	31/65 (47.7)	23/56 (39.65)	NA	NA	NA	NA	NA
Hu Yun et al. [47]	China	8/32 (25)	NA	NA	NA	NA	NA	NA
Yun Ling et al. [48]	China	55/66 (82)	NA	NA	NA	4/58 (7)	NA	NA
Youjiang Li et al. [49]	China	5/13 (38)	1/13 (7)	5–15	15	NA	NA	NA
Yuhan Xing et al. [50]	China	3/3 (10)	NA	14	28	NA	NA	NA
Yongjian Wu et al. [51]	China	41/74 (55)	NA	28	11	NA	NA	NA
Fengting Yu et al. [52]	China	80/116 (69)	3/78 (4)	NA	NA	NA	17,429 ± 6920 copies/test	34–38
Guangchang Pei et al. [53]	China	NA	108/333 (32.4)	NA	NA	0/251	NA	NA
Liang Peng et al. [12]	China	2/9 (22)	1/9 (11)	NA	NA	1/9 (11)	3.22 × 10^2^–5.42 × 10^4^	NA
Tomohiro Hosoda et al. [54]	Japan	1/1 (100)	NA	15	NA	NA	200 copies/well	NA
Chen Chen et al. [55]	China	22/133 (16)	NA	NA	13	NA	NA	NA
Siew C Ng et al. [56]	China	21/21 (100)	NA	NA	NA	NA	2 × 9–7 × 1 log10 copies/mL	NA
Mi Seon Han et al. [57]	Korea	2/2 (100)	NA	5–17	18-		1.7 × 10^6^–4.1 × 10^7^ copies/mL	NA
D. Paoli et al. [58]	Italy	0/1 (0)	NA	NA	NA	0/1	NA	NA
Kelvin Kai-Wang To et al. [59]	China	4/23 (17)	2/23 (8)	NA	NA	NA	NA	NA
Liang Su et al. [60]	China	5/9 (55)	NA	NA	NA	NA	NA	NA
Yifei Chen MD et al. [11]	China	28/42 (67)	7/42	7–13	6–10	NA	NA	NA
ZhoujiePeng et al. [61]	China	0/1 (0)	NA	NA	NA	NA	NA	NA
Shufa Zheng et al. [62]	China	59/96 (59.3)	10/96 (10)	7–28	21–28	NA	3.8–5 log10 copies/mL	NA
Yang Pan et al. [63]	China	2/2 (100)	NA	5–6	NA	NA	10^4^ to 10^7^ copies/mL	NA
Roman Wölfel et al. [64]	Germany	6/9 (67)	NA	NA	NA	NA	7.00 × 10^6^ copies/mL	NA
Jie Wang et al. [65]	China	1/2 (50)	NA	NA	NA	NA	NA	21.28
Yu-Han Xing et al. [66]	China	2/3 (33.3)	NA	9	8-20	NA	NA	NA
Wang-Da Liu et al. [67]	China	1/1 (100)	NA	NA	NA	NA	NA	NA
Qing Cao et al. [68]	China	1/1 (100)	NA	15	NA	0/1	NA	NA
Fang Liu et al. [69]	China	0/1 (0)	NA	NA	NA	NA	NA	NA
Ryota Hase et al. [70]	Japan	1/1 (100)	1/1 (100)	5	NA	NA	NA	NA
Tao Zuo et al [71]	China	7/15 (46)	1/15 (6)	NA	6	NA	>3.2 × 10^4^ copies/mL	NA
Li-juan Mao, et al [72]	China	1/1 (100)	0/1 (0)	NA	NA	NA	NA	NA
Sarah Catherine Walpole et al [73]	UK	1/1 (100)	NA	NA	NA	NA	NA	NA
Alvaro Mesoraca et al [74]	Italy	6/15 (40)	NA	NA	NA	NA	NA	NA
Divjot S. Kumar et al. [75]	Colombia	0/1 (0)	1/1 (100)	NA	NA	NA	NA	NA
Hye Won Jeong et al. [76]	Korea	3/5 (60)	NA	NA	NA	NA	1.08 ± 0.16–2.09 ± 0.85 log10 copies/mL in urine 1.17 ± 0.32 log10 copies/mL in stool	NA
Garrett A.Perchetti et al. [77]	United States	20/20 (100)	NA	NA	NA	NA	NA	37.1–37.8
Xiaorong Wang et al. [78]	China	3/3 (100)	NA	NA	40	NA	NA	NA
Yuanyuan Yu et al. [79]	China	1/2 (50)	NA	NA	NA	NA	NA	NA
Fei Xiao et al. [80]	China	12/28 (42)	NA	NA	NA	NA	NA	NA
Wenjun Du et al. [81]	China	7/10 (70)	NA	9	34	NA	NA	NA
Maksim Kirtsman MDCM et al. [82]	Canada	1/2 (50)	NA	NA	NA	NA	NA	NA
Jiufeng Sun et al. [83]	China	171/490 (34)	NA	NA	NA	NA	NA	NA
Bao Fu et al. [84]	China	1/1 (100)	NA	NA	NA	NA	NA	NA
Soo-kyung Park et al. [85]	Korea	2/46 (4) case 12/213 (5) sample	7/46 (15)	50	NA	NA	NA	29.94
Jeong-Min Kim et al. [86]	Korea	15/74 (20)	NA	NA	NA	NA	79 ± 30 copy/μL for urine and 3176 ± 7208 copy/μL for stool	NA
Amani Mansour et al. [87]	Beirut	0/1 (0)	NA	NA	NA	NA	NA	NA
Rigamonti Elia et al. [88]	Switzerland	0/1 (0)	NA	NA	NA	NA	NA	NA
Seung-Man et al. [89]	Korea	(1/1) (100)	(1/1) (100)	42	121	0/1	NA	33
Binder et al. [90]	United States	3/12(25)	NA	NA	NA	NA	NA	NA
Han et al. [91]	South Korea	10/12(83)	NA	21	7	2/12 (17) 3.82–7.55 log10 copies/mL	4.1–10.27 log10 copies/mL	NA
Colavita et al. [92]	Italy	2/2 (100)	NA	15–17	3	0/2	NA	30.1–38.59
Slaats et al. [93]	The Netherlands	1/1 (100)	NA	42	23	NA	NA	NA
Italiano et al. [94]	United States	1/1 (100)	1/1 (100)	21	7	0/1	NA	NA
Scutari et al. [95]	Italy	2/2 (100)	NA	NA	NA	1/2 (50)	6–52 copies/mL for N	NA
Hinojosa-Velasco et al. [96]	Mexico	1/1 (100)	NA	NA	NA	NA	NA	NA
Zhang et al. [97]	China	93/258 (36)	47/82 (57.3)	28–35	NA	NA	NA	CT: 19–30
Wang et al. [98]	China	20/69 (28)	NA	25	9	NA	NA	CT: 25–27
Npvazzi et al. [99]	Italy	11/107 (10.3)	NA	NA	NA	1/85 (1.2)	4.1 × 10^6^ copies/ml	NA
Chen et al. [100]	China	1/4 (25)	NA	100	NA	NA	NA	NA
Khoury et al. [101]	United States	1/1 (100)	NA	NA	NA	NA	NA	NA
Chen et al. [102]	China	52/97 (53.61)	6/97 (6.19)	NA	14.13 ± 8.61	NA	NA	NA
Barth et al. [103]	China	(50)	NA	NA	NA	NA	NA	NA
Fumian et al. [104]	United States	33/121 (28)	NA	NA	NA	NA	NA	NA
Chu et al. [105]	China	1/1 (100)	NA	NA	NA	NA	NA	NA

**Table 2:** Selected data extraction fields employed in the literature review protocol.

Study	Country	Site of Sampling	Sample Size	Virus Concentration Method	Virus Detection Method	Quantitative Data
Wurtzer S et al. [106]	France	three wastewater treatment plants	NA	centrifugation and filtration step	RT-qPCR	5.10^4^ to 3.10^6^ GU/L
Warish Ahmed et al. [15]	Australia	suburban pumping station andurban wastewater treatment plants	100–200 mL	direct RNA extraction from electronegative membranes and ultrafiltration	RT-qPCR	1.9–12 copies/100 mL
Bilge Alpaslan Kocamemi et al. [107]	Turkey	wastewater treatment plants and manholes	250 mL	Amicon^®^ Ultra-15 with 10 KDa cutoff ultrafiltration units	RT-qPCR	2.89 × 10^3^–9.33 × 10^4^ (Virus titer/liter)
Sara Giordana Rimoldi et al. [108]	Italy	rivers and wastewater treatment plants	NA	glass fiber filtration	RT-PCR-cell culture	NA
Gertjan Medema et al. [17]	The Netherlands	wastewater treatment plant	250 mL	centrifugation and filtration step	RT-PCR	NA
Walter Randazzo et al. [109]	Spain	WWTPs	NA	centrifugation and filtration step	RT-qPCR	5.38 ± 0.21 log genomic copies/L
Wu FQ et al. [110]	United States	urban wastewater	NA	centrifugation and filtration step	qPCR	6 log units
Artem Nemudryi et al. [111]	United States	pre-treated wastewater	500 mL	filtration	RT-qPCR	NA
Willemijn Lodder et al. [112]	The Netherlands	airport wastewater	10 L	NA	RT-PCR	NA
Wurtzer S et al. [113]	France	wastewater treatment plant	11 mL	ultracentrifugation	RT-PCR	NA
Manupati Hemalatha [114]	India	raw sewage	100–500 mL	filtration	RT-qPCR	30,818–266,360 copies/L
Kyle Curtis [115]	England	raw and treated wastewater	50 mL of raw wastewater 200 mL of treated final effluent	electronegative filtration	RT-ddPCR	159 copies/100 mL
Pei-Ying Hong [116]	Saudi Arabia	hospital wastewater	250 to 500 mL	HA membrane and filtration	qPCR	6.89-log copies/L
Hyatt Green [117]	Syracuse	wastewater treatment plants, influent pump stations, or interceptor lines	1.9 L	ultracentrifugation	RT-qPCR	42.7 genomes/mL
Salmaan Sharif [118]	Pakistan	raw sewage	500 mL	centrifuged	RT-qPCR	NA
Rimoldi et al. [119]	Italy	raw and treated wastewater	NA	filtration	RT-PCR	NA
Medema et al. [120]	The Netherlands	sewage	250 mL	ultrafiltration	RT-PCR	2.6–2.2 × 10^3^
Prado et al. [121]	Brazil	raw sewage	NA	NA	RT-qPCR	Ct: 36.3–39.8.
SP Sherchan et al. [122]	United States	untreated and treated wastewater	1 L	ultrafiltration, and an adsorption–elution method using electronegative membranes	RT-qPCR	3.2 log10 copies/L from the N1 and 2.5 and 3.0 log10 copies/L from the N2 assay
Kumar et al. [75]	India	wastewater treatment plant	50 mL	filtration and poly ethylene glycol (PEG) methods	PCR	5.6 × 10 3.5 × 10^2^ copies/L
Trottier et al. [123]	France	wastewater upstream of the main wastewater treatment plant	50 mL	centrifugation and 40 μm cell strainer	RT-qPCR	NA
Mlejnkova et al. [124]	Czech Republic	wastewater treatment plant	500 mL	flocculation using beef extract solution	RT-qPCR	Cq: 34–40
Haramoto et al. [125]	Japan	influent and secondary-treated wastewater	1 L	electronegative membranevortex (EMV) prior to the filtration	RT-qPCR	1.4 × 10^2−2.5^ × 103 copies/L
Rosa et al. [126]	Italy	raw sewage	250 mL	PEG dextran	RT-PCR	NA
Gonzalez et al. [127]	United States	wastewater treatment plant	1 L	filtration	RT-ddPCR	10^1^–10^4^ copies/100 mL
Miyani et al. [128]	United States	wastewater treatment plant	1 L	NanoCeram electropositive cartridge filters	RT-PCR	10^4^–10^5^ genomic copies/L
Westhaus et al. [129]	Germany	wastewater treatment plant	45 mL	centrifugation and filtration steps	RT-PCR	3–20 gene equivalents/mL in the inflow, and 2.7–37 gene equivalents in the effluent
D’Aoust et al. [130]	Canada	Influent post grit solids and primary clarified sludge in two water resource recovery facilities	6 L	1.5 µm glass fiber filter followed by a 0.45 µm GF6 mixed cellulose ester filter	RT-qPCR and RT-ddPCR	1.7 × 10^3^ to 3.8 × 105 copies/L
Hata et al. [131]	Japan	wastewater treatment plants	80 mL	polyethylene glycol precipitation	TaqMan-based qRT-PCR	1.2 × 10^4^–3.5 × 10^4^ copies/L
Jafferali et al. [132]	Sweden	untreated municipal wastewaters	NA	ultrafiltration, double ultrafiltration, adsorption-extraction, centrifugation combined with adsorption-extraction	RT-qPCR	Cq: 35.71–38.15
Hasan et al. [133]	United Arab Emirates	influents and treated effluents of wastewater treatment plants and untreated wastewater	250 mL	ultrafiltration	RT-qPCR	2.86 × 10^2^–3.4 × 10^4^ copies/L
Baldovin et al. [134]	Italy	municipal wastewater treatment plant	1000 mL	ultrafiltration	RT-qPCR	4.8-4.9 log10 gc/L
Feng et al. [135]	China	flushing in the toilet and in sewage pipes	15 mL	NA	RT-qPCR	3092 copies/mL in sewage/wastewater
Kumar et al. [136]	India	wastewater treatment plants	NA	filtration and polyethylene glycol precipitation	RT-qPCR	Ct: 22–37
Saguti et al. [137]	Sweden	influent and effluent wastewater treatment plant	500 mL	adsorption to milk powder and to Nano-Ceram filter	RT-qPCR	0.14–16 log10/L
Gallardo-Escárate et al. [138]	Southern Chile	untreated wastewater	10 L	filtration and centrifugation	RT-PCR	4 × 10^3^–7 × 10^3^ Genome Unit/L
Gonçalves et al. [139]	Slovenia	hospital wastewater	1 L	filtration	RT-PCR	Ct: 29.65–37.03 for RdRP, 33.61–38.65 for E
Martin et al. [140]	England	sewage plant	1 L	filtration–centrifugation	RT-qPCR	3.50 and 4.20 Log10 gc/L
Peccia et al. [141]	United States	primary sewage sludge	40 mL	-	qRT-PCR	1.7 × 10^3^ mL^−1^ to 4.6 × 10^5^ mL^−1^
Albastaki et al. [142]	United Arab Emirates	effluent of wastewater treatment plant	1 L	filtration–centrifugation	RT–PCR	Ct: 32–36
Agrawal et al. [143]	Germany	wastewater treatment plants	NA	filtration	RT-PCR	3 × 10^13^–2 × 10^14^ copies/day
Abu Ali et al. [144]	Israel	wastewater treatment plants	NA	ultrafiltration	RT-qPCR	7 × 10^3^ copies/mL
Kocamem et al. [145]	Turkey	influent, effluent, primary and waste activated sludge	250 mL	filtration–centrifugation then PEG	RT-qPCR	2.6 × 10^2^–8.2 × 10^6^ titers/L
Al et al. [146]	Canada	sewer shed site and influent	100 mL	centrifugation	RT-qPCR	6.6 × 104–1.4 × 104 GU L-1
Iglesias et al. [147]	Argentina	raw water samples	500 mL	centrifugation and PEG	RT-qPCR	NA
Wu et al. [148]	China	raw sewage	NA	filtration	RT-PCR	<90 copies/mL
Lara et al. [149]	The Netherlands	sewage	100–200 mL	ultrafiltration	RT-qPCR	Ct: 32.5–34.6
Garham et al. [150]	United States	influent and primary settled solids	50 mL	PEG precipitation	ddPCR and RT-QPCR	730–4250 cp/g for N1
Hokajärvi et al. [20]	Finland	influent of wastewater treatment plant	2–5 L	ultrafiltration	RT-qPCR	8.1 log10 copies 100 mL^−1^
